# Reconstruction of fire regimes through integrated paleoecological proxy data and ecological modeling

**DOI:** 10.3389/fpls.2014.00785

**Published:** 2015-01-22

**Authors:** Virginia Iglesias, Gabriel I. Yospin, Cathy Whitlock

**Affiliations:** ^1^Montana Institute on Ecosystems, Montana State UniversityBozeman, MT, USA; ^2^Department of Earth Sciences, Montana State UniversityBozeman, MT, USA

**Keywords:** fire, charcoal, reconstruction, modeling, paleoecology

## Abstract

Fire is a key ecological process affecting vegetation dynamics and land cover. The characteristic frequency, size, and intensity of fire are driven by interactions between top-down climate-driven and bottom-up fuel-related processes. Disentangling climatic from non-climatic drivers of past fire regimes is a grand challenge in Earth systems science, and a topic where both paleoecology and ecological modeling have made substantial contributions. In this manuscript, we (1) review the use of sedimentary charcoal as a fire proxy and the methods used in charcoal-based fire history reconstructions; (2) identify existing techniques for paleoecological modeling; and (3) evaluate opportunities for coupling of paleoecological and ecological modeling approaches to better understand the causes and consequences of past, present, and future fire activity.

## Introduction

Fire has been an integral part of the Earth system for the past 350 million years (Scott and Glasspool, 2006). Today, it is the dominant disturbance agent in most terrestrial ecosystems (Bowman et al., 2009), and its legacy effects–especially in forests–may persist for centuries or millennia. Projected changes in fire are expected to trigger biotic reorganizations with broad consequences for land-surface feedbacks and the global carbon cycle (Flannigan et al., 2009; Parisien and Moritz, 2009). Such scenarios will profoundly impact ecological services ranging from water availability to nutrient cycling and recreation, and have an economic, esthetic, and cultural cost.

The fire regime (i.e., the characteristic frequency, size, and intensity of fire) results from complex interactions between long-term trends in climate and local fuel availability and probability of ignition (Heyerdahl et al., 2008). In most parts of the world, human use and control of fire has led to fire regimes that reflect not only natural processes but also social-economic drivers and their evolution through time (Bowman et al., 2011). Thus, fire regimes can only be understood through the assessment of climate-vegetation-human-fire interactions at a variety of temporal and spatial scales (Whitlock et al., 2010; Moritz et al., 2012).

Paleoecological studies offer information on long-term fire variability and its relationship with a broader array of environmental conditions than can be observed at present. Such information is central to providing a context for modern and future fire activity and helping to identify the causes and consequences of fire at local-to-global scales. Robust assessments of environmental change, however, require the incorporation of quantitative measurements and uncertainty estimation to the typically qualitative nature of paleoecological studies.

**Figure 1:**
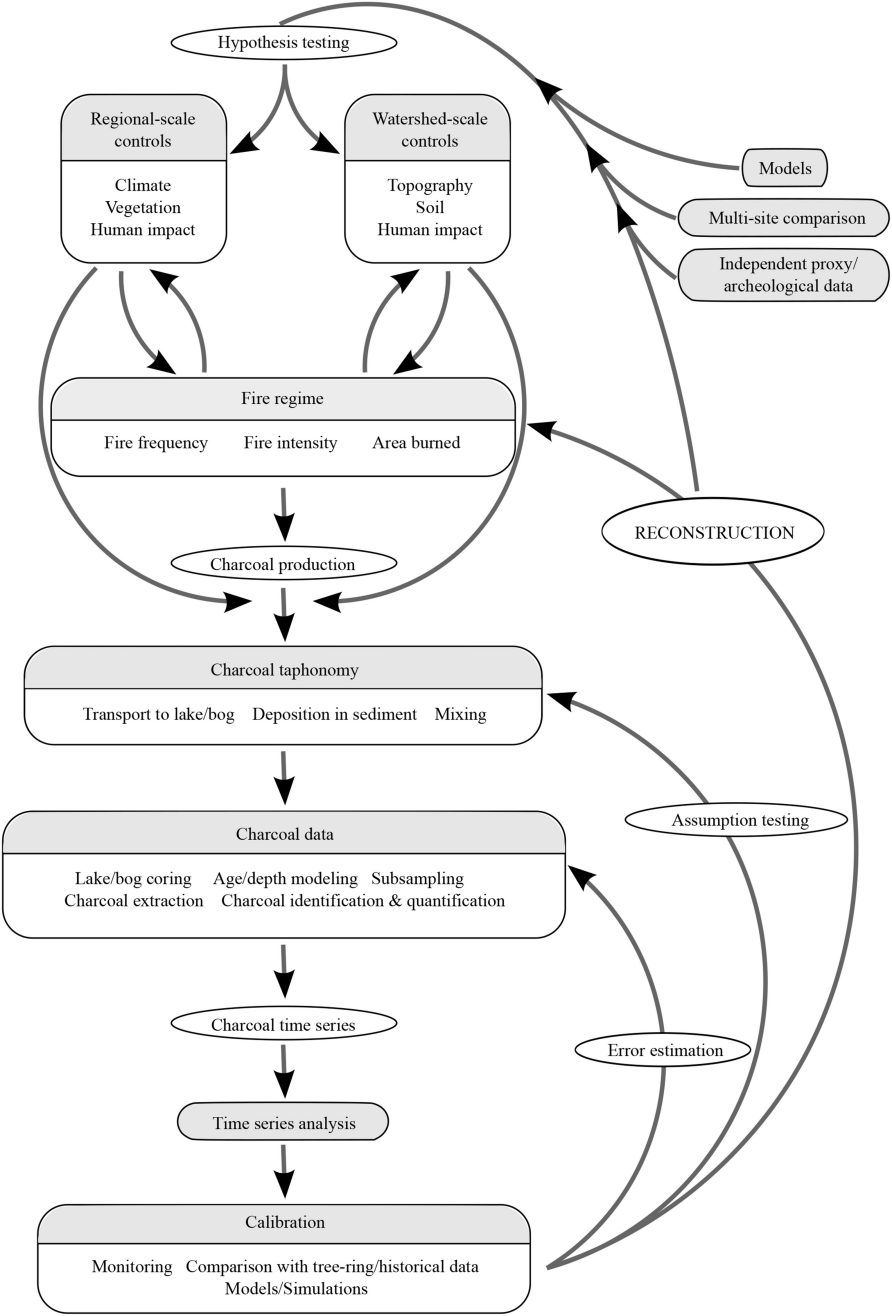
**Reconstructing fire histories from lake sediments.** Upper-left: charcoal production and taphonomy (i.e., natural sources of variability in the charcoal record). Lower-left: the charcoal record (i.e., analytical sources of variability in the charcoal record). Right: Steps in the reconstruction of past fire activity.

An integration between paleoecology and ecological modeling is thus needed to fully understand climate-vegetation-human-fire linkages (Davis, 1994; Anderson et al., 2006; Peng et al., 2011). Recent studies exemplify integrative research that effectively combines both disciplines (Table 1; e.g., Ganopolski et al., 1998; Heiri et al., 2006; Bradshaw, 2008; Brubaker et al., 2009; Colombaroli et al., 2010; Henne et al., 2013; Brücher et al., 2014). These studies demonstrate the power of empirical paleoecological records to describe different components of past fire regimes, and the use of modeling approaches and data-model comparisons to offer broader inference of top-down drivers of past fire activity. In this manuscript, we (1) review the use of sedimentary charcoal as a fire proxy and the methods used in charcoal-based fire history reconstructions; (2) identify existing techniques for paleoecological modeling; and (3) evaluate opportunities for coupling of paleoecological and ecological modeling approaches to better understand the causes and consequences of past, present and future fire activity (Figure 1).

**Table 1:**
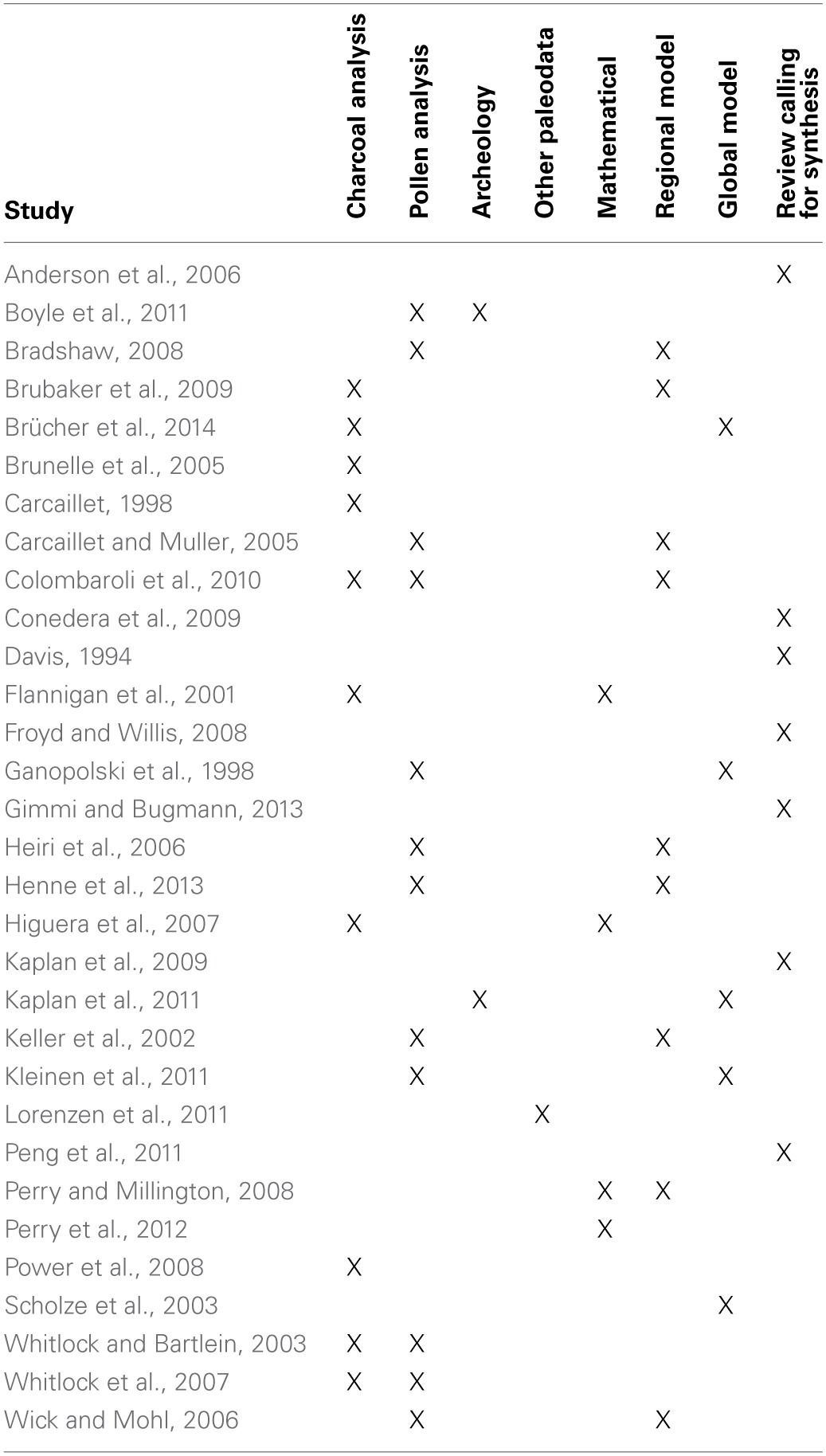
**Studies utilizing a combination of paleoenvironmental data and ecological modeling mentioned in this paper**.

## Charcoal data and related modeling techniques

Information on past fire activity comes from a variety of sources, including geochemistry, tree-rings, charcoal, archeology, and written documents. We focus on sedimentary charcoal analysis and, in particular, the study of lake- and bog-sediment records, because it has become the most widely used approach for high-resolution reconstructions spanning millennia. Charcoal is formed from the incomplete combustion of fuel under reducing conditions. Charcoal particles are carried aloft during the fire, transported to lakes by airborne and surficial processes, and incorporated into the sediments where they are preserved. Year by year, lake and bog sediments collect and bury charcoal from local and distant fires, along with pollen, macrofossils, and diatoms (Whitlock and Larsen, 2001; Conedera et al., 2009). Sediment cores are thus excellent repositories of paleofire information, and analysis of the charcoal, pollen, lithology, and geochemical components preserved in sediments is widely used to reconstruct watershed history, as well as the changing character of climate-vegetation-fire linkages.

Charcoal analysis is based on the assumption that stratigraphic intervals with abundant charcoal particles represent a primary contribution that is deposited mainly through aerial fallout during or shortly after a fire (Whitlock and Larsen, 2001). Temporal changes in charcoal abundance are inferred from downcore variability in charcoal concentrations. Changes in sedimentation rates through time affect the interpretation of charcoal concentration data, and charcoal accumulation rates (CHAR; charcoal particles ^*^ sediment volume^−1 *^ time^−1^) are generally considered a better proxy of past fire activity (Long et al., 1998).

Since the early reviews of Tolonen (1978) and Patterson et al. (1987), charcoal-based studies have become a primary source of information about long-term fire dynamics in most terrestrial ecosystems. Today, well-dated charcoal records are available for most parts of the world. For example, charcoal records from South America suggest that fire is a natural component of arid (Paduano et al., 2003), temperate and cool-temperate (Huber et al., 2004; Whitlock et al., 2007; Markgraf et al., 2013) and tropical wet ecosystems (Cordeiro et al., 2008), and changes in past fire activity have been linked to climate variability in Asia (e.g., Sun et al., 2000), Africa (Mworia-Maitima, 1997), Oceania (Black and Mooney, 2006), Europe (Daniau et al., 2007; Zumbrunnen et al., 2009), and the Americas (Behling, 1997; Moreno, 1997).

The interpretation of sedimentary charcoal data is not straightforward. Stratigraphically ordered sampling (i.e., temporal autocorrelation), non-random data, non-linear trends, uneven sampling over time and heterogeneous variance pose methodological and statistical challenges to the analysis of time series data. Moreover, processes affecting charcoal production, transport, and deposition and issues associated with the creation of an independent radiometric chronology compound uncertainties in fire history reconstructions. Assessment of climate-vegetation-fire dynamics therefore requires clever experimental designs, cautious inspection of the data and refined statistical analyses. Modeling can effectively address all of these requirements because it can fill in the information gaps inherent in paleoecological data and contribute to a mechanistic understanding of charcoal taphonomy, fire behavior, and fire-vegetation dynamics. We highlight several studies that meet these criteria throughout this review.

Ecological models incorporate varying degrees of complexity, resolution, and processes to portray fire-vegetation-climate interactions. Modeling approaches may be conceptual, statistical, or mechanistic. Conceptual models are based on sets of logical rules, and are often used where processes are complex, poorly understood, or both. Statistical models rely on empirical measurements, and often reflect a better understanding of the processes being modeled. Finally, mechanistic models incorporate equations that describe individual processes, implying a high degree of understanding of the system. Examples of these three approaches within the field of small-scale fire modeling include conceptual models like the USDA Forest Service's standard fuel models (Scott and Burgan, 2005), statistical models such as Rothermel's (1972) derivation of fire spread rates, and sophisticated and computationally demanding fluid dynamic modeling of fire spread (Dupuy and Morvan, 2005). These degrees of model complexity are evident in larger-scale fire models as well, such as those designed to simulate fire over landscapes. Statistical correlations between fire weather and area burned in the recent past have been used to reconstruct the size and distribution of fires during the Holocene, and to predict future area burned (Flannigan et al., 2001; Bergeron et al., 2006). Statistical modeling has also been used to simulate the production, transport, and deposition of charcoal during and following fires (Higuera et al., 2007), and these insights have helped justify the use of charcoal data as a proxy for past fires.

Models may include greater degrees of complexity by allowing some of the data and conditions that drive the model to update during the course of a simulation based on the ongoing conditions within the model. These “dynamic” (as opposed to “stationary”) ecological models utilize interacting modules, each of which simulates different ecosystem properties and processes (e.g., Sitch et al., 2003) to extrapolate from micro- (e.g., carbohydrate production during photosynthesis) to macro-scale dynamics (e.g., global carbon cycling). Such models can increase understanding of complex, emergent phenomena (Perry and Millington, 2008).

Dynamic global vegetation models (DGVMs) are a class of dynamic models used to simulate shifts in potential vegetation at the regional to global scale as a response to shifts in climate. DGVMs simulate biogeochemistry, hydrology, vegetation, and disturbance, by attempting to explicitly represent processes like photosynthesis, plant competition, and mortality. Most DGVMs include several mechanistic modules, while still relying on some statistical and conceptual modules. Simulations develop an “equilibrium” vegetation that establishes initial values for various components, and experiments are then run in a spatially distributed mode across thousands of grid cells, each of which is assumed to have relatively homogeneous conditions. DGVMs allow the conditions from a prior time-step to strongly influence the system response at the current time-step, which makes possible the representation and description of changing ecosystem properties like a disturbance regime. DGVMs with interactive fire components describe biomass burning at regional to global scales by considering climate, ignition, surface fire spread, fire behavior, post-fire mortality, and carbon emissions to estimate area burned and biomass burned over the simulation area (Thonicke et al., 2010; Pfeiffer et al., 2013).

Because paleoecological studies are often used to infer information at the scale of landscapes, there are substantial benefits to research that integrates paleoecology with models that are intended to function at the landscape scale. At this scale, successful models usually include components that are conceptual, statistical, and mechanistic. Modules that are more conceptual within one landscape modeling system may be more mechanistic within another landscape modeling system. For example, seed production is more conceptual within LANDIS-II (Scheller et al., 2007), due to a low level of specificity with regard to plant community composition, whereas it is more mechanistic in FireBGCv2, which tracks more detailed information about individual trees (Keane et al., 2011). The variety of approaches in landscape models can be especially useful in systems where prehistoric human land use and anthropogenic burning are difficult to infer from the paleoecological record. Modeling approaches that range from stationary and largely conceptual (e.g., Perry et al., 2012) to those that are dynamic and largely mechanistic (e.g., Colombaroli et al., 2010) have been useful in evaluating competing hypotheses regarding the role of prehistoric humans in shaping the fire regime and vegetation.

Paleoecological reconstructions often motivate particular modeling experiments, and data, in turn, are used to validate model simulations of past conditions (e.g., COHAMP members, 1998; Bartlein et al., 2011). Data-model comparisons constitute a powerful tool for understanding particular processes (Higuera et al., 2011) as well as exploring explanations for past variations in vegetation and biomass burning (Daniau et al., 2012; Marlon et al., 2013; Molinari et al., 2013). Such comparisons require identifying the scales and precision inherent in the research question, understanding the resolution and uncertainties inherent in the data, and selecting a model in which the degree of integration, number of processes explicitly simulated, and temporal and spatial resolution are appropriate to the question (Bartlein and Hostetler, 2004). Even in cases where the assumptions embedded in charcoal analysis prevent direct quantitative comparisons with modeled output, non-parametric techniques can be employed in model-data comparisons (e.g., Brücher et al., 2014).

## Local fire activity: charcoal source area

Time series of sedimentary charcoal abundance have become a widely used tool for reconstructing long-term trends in biomass burning. Interpretation, however, rests on understanding the source area of charcoal particles (i.e., the area burned and distance to the site) and the ability to accurately infer other properties of the fire regime, such as frequency, intensity, and fuel type. Constraining the charcoal source area is not a trivial task because charcoal abundance is influenced by vegetation, fire climate, and fire weather as well as site-specific processes related to charcoal deposition and burial. Clark (1988) used particle physics to model the aerial transport and deposition of charcoal particles. In the model, a distance-decay function was used to predict larger transport distances for increased wind speeds and smaller particles. Subsequent calibration studies focused on monitoring the incorporation of charcoal in lake sediments after recent fires partially support Clark's model. In the International Crown Fire Modeling Experiment (ICFME) in the boreal forest of Siberia, for example, Lynch et al. (2004) reported high deposition rates close to the fire and an abrupt decrease in charcoal abundance at farther distances (i.e., >10 km), which matched the Clark (1988) model predictions of non-linear charcoal deposition pattern. Conversely, a broad range of particle sizes were observed within the boundaries of low- and high-intensity fires in the mid- and high-latitudes (Whitlock and Millspaugh, 1996; Pitkänen et al., 1999; Asselin and Payette, 2005), suggesting that particle size alone was not a reliable prediction of transport distance.

Correlation between charcoal records and known fire events (e.g., Gardner and Whitlock, 2001) indicate that charcoal from long distances can comprise a significant proportion of the total amount of particles. Such particles tend to be small (<100 mm), whereas dispersal of large charcoal particles is strongly biased toward short distances (Peters and Higuera, 2007). Much attention has therefore turned to the use of macrocharcoal (particles >125 mm in diameter) as a proxy for local fire history. Charcoal within this relative large size class is expected to have high settling velocities and to be associated with local source areas (<20 km from the lake) (Higuera et al., 2011). Similar conclusions were reached by Oris et al. (2014) after a 3-year study to monitor charcoal dispersion and deposition in boreal lakes. Their findings suggest that both local and regional fires are associated with higher concentrations of charcoal particles of all sizes in the sediments of lakes. Charcoal assemblages resulting from regional fires, however, tend to be characterized by higher proportions of small particles, whereas large particles represent more local events. The analysis of particle size distribution thus emerges as a tool to discriminate charcoal source area.

Analyses of other paleoenvironmental proxies complement this approach and aid in the identification of local fires. A number of studies have noted the correspondence between charcoal variability and changes in the pollen of fire-adapted plants (e.g., Swain, 1973; Patterson and Backman, 1988; Pitkänen et al., 1999) and diatom taxa (Ponader et al., 2002). Local high-severity fires have indirectly been inferred from increases in varve thickness, anomalously high content of aluminum, vanadium, and silt in sediments containing abundant charcoal (Cwynar, 1987), and peaks in carbon, nitrogen (Dunnette et al., 2014), and magnetic susceptibility (Rummery et al., 1979; Rummery, 1983) associated with post-fire erosion, terrestrial nutrient loss, and topsoil oxidation. Other studies, however, find the relationship between erosion and charcoal accumulation less straightforward (Long et al., 1998) and note a complex relationship between fires and sediment transport, which is dependent not only on fire location but also on fire intensity, fuel, soil, and substrate (Meyer et al., 1995).

In spite of advances in linking fire activity and charcoal accumulation in lakes, reconstructing area burned remains a challenge with great potential for model-data integration. Such efforts have been very effective for some aspects of charcoal analysis. For example, Higuera et al. (2007) used a statistical model that incorporated size, location, and frequency of fire as well as charcoal taphonomy and sampling techniques to evaluate assumptions of charcoal analysis. Simulations of charcoal accumulation are consistent with empirical data that suggested that sedimentary charcoal is an excellent proxy of fire activity within a 1–10 km radio of the lake (Higuera et al., 2011; Kelly et al., 2013). These studies helped validate charcoal-based fire reconstructions and identify uncertainties associated with natural (e.g., charcoal production, transport, and deposition) and analytical processes.

## Local fire activity: fire frequency, severity, and fuel type

Variability in the charcoal record comes from a variety of sources, including temporal variations in primary charcoal production, transport, and deposition; bioturbation and addition of secondary (remobilized) charcoal; and differences in sampling, chemical treatment, and charcoal identification and quantification. These sources can obscure the relationship between charcoal accumulation rates (i.e., CHAR) and fire. Consequently, interpretation of the charcoal time series usually involves decomposing the record into low- and high-frequency components (Higuera et al., 2005). The low-frequency component, also known as background charcoal, has been linked to regional charcoal production (Whitlock and Millspaugh, 1996) and long-term changes in charcoal delivery mechanisms before its inclusion in lake sediment (Long et al., 1998). More recent calibration studies in Yellowstone National Park and the boreal forests of Alaska suggest correlations of background CHAR and area burned within a radius of <30 km from the lake (Higuera et al., 2011; Kelly et al., 2013). Conversely, positive anomalies in the high frequency component of the charcoal time series (i.e., charcoal peaks) result from local fire episodes (i.e., one or more fires occurring within a few km of the lake during the deposition time) (Higuera et al., 2010).

Time and frequency domain approaches are used to decompose the charcoal time series into background and peaks (Clark and Royall, 1996), thus accounting for long-term changes in charcoal production and allowing a more sophisticated reconstruction of the fire history. In most studies, background charcoal is defined by fitting non-parametric smoothers to the charcoal time series (Higuera et al., 2007; Higuera, 2009; Blarquez et al., 2013). The positive residuals of the model (i.e., charcoal peaks) are inferred to represent fire episodes as well as natural and statistical noise. A user-defined threshold is frequently applied to the charcoal peaks to separate fire episodes from noise. Although based on poorly understood assumptions regarding charcoal taphonomy (MacDonald et al., 1991; Whitlock and Millspaugh, 1996), fire reconstructions following this method have been shown to correspond well with historical (Mensing et al., 1999; Kelly et al., 2013) and dendrochronological records of local fires (Higuera et al., 2005). However, as noted by Higuera et al. (2010), not all charcoal records are suited for peak detection, and in many cases the signal-to-noise ratio is too low to detect significant charcoal peaks from background trends. As a result, the limitations and assumptions of each fire history reconstruction should be assessed and clearly stated.

Identifying the location (i.e., local vs. regional) and time of individual fire events allows estimation of changes in fire frequency (i.e., number of fire events per unit time) and fire return interval (i.e., years between fire events) over millennia. Assuming that CHAR time series represent trends in total biomass burned, Ali et al. (2012) proposed the CHAR-to-fire frequency ratio as a semi-quantitative proxy of fire severity. Reconstruction of long-term trends in fire frequency and severity aids in the understanding of the temporal and spatial variability of fire and its impact on landscape heterogeneity.

In addition to fire frequency (fire return intervals) and severity, additional information about past fires can be inferred from the identification of the charcoal particles. Umbanhowar and Mcgrath (1998), for example, used charcoal morphology as an indicator of type of biomass burned and fire intensity. A similar approach allowed the identification of changes in the dominant fuel (i.e., grass or wood) in Patagonia (Whitlock et al., 2006; Iglesias et al., 2012) and tropical Africa (Aleman et al., 2013). Vannière et al. (2003) analyzed the structure and morphology of charred particles from soil samples and noted distinguishing characteristics between airborne and reworked charcoal. Based on this idea, Enache and Cumming (2007) used morphological features in charcoal particles to infer taphonomic processes, and interpreted the presence of fragile particles as an indicator of fires in close proximity to the study site.

Anthracological studies (i.e., analyses of the anatomical structure of charred fragments in soils and archeological sites) have allowed the identification of the botanical origin (i.e., taxon) of large charcoal particles (>250 μm), as well as information on the structural characteristics (i.e., branch vs. trunk) and state (i.e., dead vs. alive) of the fuel before carbonization (e.g., Bégin and Marguerie, 2002; Marguerie and Thibaudeau, 2004; Allué et al., 2005; Asouti and Austin, 2005; Dufraisse, 2006; Carcaillet, 2007; Braadbaart and Poole, 2008; Thery-Parisot et al., 2010). Charcoal-based fire history reconstructions would greatly benefit from the application of techniques used in anthracolology to better characterize changes in fuel composition and fire intensity.

## Regional fire activity: climate-vegetation-fire linkages

Fires are complex ecological processes. Hierarchy theory states that ecological processes are driven by factors acting at different scales, in which higher-level spatial factors constrain the effects of more local ones to various degrees (Turner, 2001). Studies of past biomass burning are grounded in this conceptual understanding of top-down and bottom-up influences on fire occurrence, and the interpretation of past fire activity often focuses on disentangling broad-scale slowing varying drivers, such as climate and vegetation, from local controls related to site characteristics, fuel conditions, and human activity (Whitlock et al., 2010).

The interactions between fire and climate are the most direct. Dendroecological studies, for example, have shown that changes in large-scale atmospheric circulation and seasonal weather patterns synchronize fire activity across broad regions by affecting the length of the fire season and the moisture content of fuels (Swetnam, 1993; Heyerdahl et al., 2001; Kitzberger et al., 2012). Similarly, composites of charcoal time series across continents reveal trends in biomass burning related to millennial scale variations in seasonal cycle of insolation and ocean-land interactions (Carcaillet et al., 2002; Power et al., 2008, 2013; Daniau et al., 2012; Marlon et al., 2012). In both the northern hemisphere (Daniau et al., 2007) and Australasia (Mooney et al., 2011), fire activity matches Dansgaard-Oeschger cycles observed in ice cores from Greenland, indicating long-term associations between regional-to-continental-scale biomass burning and warming. Additionally, a comparison of charcoal records from Indonesia, Papua New Guinea, Central and South America shows that fire is not only dependent on mean atmospheric conditions but also on hemispheric climate teleconnections that define interannual variability (Haberle and Ledru, 2001).

At the regional-scale, comparison of charcoal records between adjacent watersheds has been used to identify the importance of climate in synchronizing fire activity (Gavin et al., 2006; Walsh et al., 2010). Synchronous shifts in CHAR across multiple sites (Gavin et al., 2003; Rius et al., 2011) have been attributed to changes in regional fire activity related to periods of warmer temperature and decreased effective moisture. Nonetheless, multi-site comparison of fire histories also suggests that the climate-fire relationship is not linear, and the relative importance of regional- vs. watershed-scale controls of ecosystem dynamics changes as critical thresholds are reached (Ali et al., 2009; Iglesias et al., 2012). At present, for example, topography and fuel type effectively limit fires in the coastal temperate rainforest of western North America (Gavin et al., 2003) and the Alps (Carcaillet et al., 2009). During the early Holocene, however, when Northern Hemisphere summer insolation was higher than present, warmer fire-season temperatures and extended droughts synchronized fire activity in both regions. Thus, cross-scale studies reveal that the fire regime is the product of non-stationary interactions of large- and small-scale drivers of ecosystem dynamics (Turner and Romme, 1994).

Charcoal and pollen data show that vegetation influences fire regimes by affecting the type, load and spatial distribution of fuel across the landscape. In humid regions, dense woody vegetation provides abundant and continuous fuel. However, due to the high water content of plant tissues and soil, protracted droughts are necessary for fuel to become flammable and fires to spread. Thus, as exemplified by the fire history of Xishuangbanna in southern China (Gu et al., 2008), infrequent high-intensity fires in tropical rainforests are strongly controlled by fuel moisture.

Conversely, fine fuels in seasonally-xeric environments desiccate quickly and are frequently dry enough to support fires, even during non-drought years. Areas where weather and climate are conducive to natural ignitions may not be those with the most active fire regimes because their climates may not support continuous flammable vegetation (Huber et al., 2004; Parisien and Moritz, 2009; Iglesias et al., 2014). In these ecosystems, frequent low-intensity fires are limited by fuel accumulation (Krawchuk et al., 2009). Postglacial composite charcoal records from the Americas (Power et al., 2008; Moreno et al., 2010; Iglesias and Whitlock, 2014), for example, show a direct correlation between moisture and fire at millennial time scales suggesting that, during glacial times, regional-to-continental-scale biomass burning was primarily limited by fuel availability, and rose during the Holocene, as vegetation density increased.

Comparison of pollen and charcoal from multiple sites shows that climate-vegetation-fire dynamics are complex and change as ecosystems evolve. In Alaska, the colonization of *Picea mariana* during the cool humid middle Holocene was associated with a pronounced increase in fire frequency, suggesting that the bottom-up controls of vegetation may not only amplify the effects of climate on fire regimes, but also dampen them (Higuera et al., 2009). Similarly, the fire history of subalpine forest in the Colorado Rockies showed little variation in fire frequency over the last 6000 years, but changes in biomass burned per fire (and inferred fire severity) indicated a response to climate-induced changes in forest density (Higuera et al., 2014).

Complex ecosystem dynamics resulting from climate-vegetation-fire feedbacks may be difficult to infer from proxy data alone and modeling efforts have helped explicitly incorporate assumptions and estimate uncertainties. Several studies have compared, and in some cases integrated, charcoal records and ecological modeling at regional-to-continental scales, allowing for an improved understanding of the evolution of the fire regime (e.g., Brubaker et al., 2009; Colombaroli et al., 2010; Molinari et al., 2013). In general, successful efforts have relied on conceptual and statistical models of fire behavior and charcoal deposition, at a range of scales from landscape to global. Flannigan et al. (2001), for instance, derived present and future fire weather maps from general circulation model and regional climate model simulations for two levels of atmospheric CO_2_ in Canada. For each of these scenarios, they generated 6000 years of fire weather, and compared the simulated paleofire weather data to charcoal data. Good agreement between the charcoal record and ignition patterns under the paleoclimate scenario lent support to their estimates of changes in area burned throughout Canada under the future climate scenarios.

More complex, dynamic representations of vegetation have also been effectively compared with fire-history reconstructions. Brubaker et al. (2009) matched fire-return intervals from a dynamic landscape ecological model (ALFRESCO) and reconstructed fire return intervals developed from three charcoal records from the southern Brooks Range, Alaska, USA, over a 100 km^2^ simulation area. They assumed a correspondence between fire occurrence in 1 km^2^ model cells and the fire episodes–as inferred from the charcoal peaks in the CHAR record—in the CHAR record's source area. Fire size and frequency were calibrated to the modern record and then validated against historic records under varying climate and vegetation scenarios. Model-data integration thus allowed for spatially explicit reconstructions of the fire regime and the quantitative characterization of the associated environmental conditions.

## Regional fire activity: the role of humans in altering the fire regime

Human use of fire likely dates back over 1,000,000 years (Berna et al., 2012) and anthropogenic impact on natural fire regimes is likely to have changed as economies shifted from hunting-gathering to pastoralism, farming, and industrialization (Pyne, 2001). Charcoal composites from around the world have been used to examine human-fire relationships at continental scales over millennia (Marlon et al., 2008). At this coarse resolution, climate emerges as the dominant driver of fire activity (Daniau et al., 2010; Mooney et al., 2011; Power et al., 2013). Additional insight has come at the local to regional scale where fire history reconstructions can be compared with archeological, ethnographic and historical records of anthropogenic burning. Cross-scale studies of biomass burning in Patagonia, for instance, show very limited human impact at both the local- and regional scales before European arrival (Iglesias and Whitlock, 2014). Conversely, striking evidence of human-induced fires and deforestation comes from charcoal and pollen records from South Island New Zealand, where initial Mâori arrival is strongly associated with widespread burning and loss of native forest (McWethy et al., 2009, 2010). Population growth during the Holocene was also associated with increased fire activity in watersheds in the Alps (Carcaillet et al., 2009), the Mediterranean basin (Vannière et al., 2011), and southeastern Australia (Black and Mooney, 2006). In eastern North America, paleoecological and ethnohistorical records of land-use reveal that human impacts were widespread but their intensity was heterogeneous (Muñoz et al., 2014).

Paleoenvironmental reconstructions thus indicate that anthropogenic impact depends on scale, prevailing climate conditions, and vulnerability of the dominant plant species to fire. Human influences are most strongly felt at the wet and dry ends of the precipitation gradient (Whitlock et al., 2010; Moritz et al., 2012; McWethy et al., 2013). At the wet end of the gradient, where biomass is high and natural ignitions are either infrequent or do not occur at the time of fuel desiccation, people have increased ignition frequency, converting closed forests to open vegetation types (e.g., Perry et al., 2012). At the dry end, fire suppression in fuel-limited landscapes has shifted vegetation to more closed, fire-prone plant communities (e.g., Staver et al., 2011).

Simulation modeling experiments play a valuable role in testing these hypotheses and assessing whether changes in climate and/or human activity were both necessary and sufficient to generate shifts in vegetation and fire under different environmental conditions. Regional vegetation modeling studies have used experimental designs that allow for the inference of humans as active agents environmental change. For example, Colombaroli et al. (2010) employed a 2 × 2 design of climate and ignition scenarios in LandClim to simulate Holocene fire activity in the Alps. By comparing the output with pollen and charcoal data, they assessed the long-term role of humans in altering the probability of fire and highlighted the risk that modern societies pose to mountain environments. Simulations have also been undertaken to infer anthropogenic impact on vegetation dynamics. Models suggest the rapid rate of deforestation reconstructed for New Zealand after the initial Mâori arrival cannot be explained by climate alone and could only have occurred if fires were targeted to the most flammable vegetation on the landscape (Perry et al., 2012). Under these conditions, 40% of forests would have been lost within decades, an estimate that matches inferences from the charcoal record (McWethy et al., 2014). Studies nested in the paleoecology-modeling interface thus serve as a context for understanding the resilience of ecosystems to natural and human-induced environmental change.

## Global fire activity

The growing geographic coverage of high-resolution charcoal data allows for reconstructions of past fire activity at continental and global scales. With the objective of archiving and sharing data for research and education, the Global Paleofire Workgroup (www.gpwg.org) supports a public-access database of fire records (e.g., charcoal, black carbon, levoglucosan) from around the world (Global Paleofire Working Group, 2014), as well as computer code for data analysis and synthesis (e.g., Blarquez et al., 2014). Compositing of charcoal records has allowed the reconstruction of biomass burning in the Americas (Marlon et al., 2009; Moreno et al., 2010) and Australasia (Mooney et al., 2011), the northern and southern hemispheres (Daniau et al., 2012) and the globe (Marlon et al., 2008; Power et al., 2008). Furthermore, composite charcoal data have been used to test hypotheses regarding the causes of large-scale environmental change. Composite records from North America, for example, suggest that the proposed asteroid impact on the Laurentide ice sheet at ca. 12,900 cal yr BP did not result in continent-wide burning (Marlon et al., 2009), as proposed by Firestone et al. (2007), but rather fire activity increased during the late-glacial to early-Holocene transition in association with rising temperatures. Long-term trends in global biomass burning have also been attributed to changes in climate, inasmuch as trends in mean temperatures correlate well with charcoal data (Daniau et al., 2012). A composite record from North American shows that during the last 200 years, however, there has been a decline in global fire activity despite favorable climate conditions (Marlon et al., 2008). In the western United States, this decline has been attributed to land cover fragmentation, fire management, and anomalous fuel accumulations (Marlon et al., 2012).

Syntheses of paleoenvironmental data disclose temporal variations in the relative amount of biomass burned at continental scales but often offer little insight into the characteristics of fire and its drivers. To tackle this issue, Brücher et al. (2014) developed a new carbon cycle model by combining existing models (i.e., Earth system carbon cycle model and land-cover carbon cycle model) and compared the output with global paleofire patterns inferred from charcoal data. Simulations under transient climate yielded fire activity that changed in concert with observed changes in the charcoal record; minor discrepancies between the simulations and the empirical data were attributed to anthropogenic influences and the effect of CO_2_ fertilization on fuel bulk density. Overall, the results of this modeling research agree with previous interpretations (Daniau et al., 2012) that show increasing fire activity in the mid to late Holocene due to changes in vegetation, although the modeling research could not indicate whether anthropogenic changes to vegetation were the ultimate driver of this increased fire activity.

## Conclusions and future directions

The use of sedimentary charcoal as a fire proxy has increased markedly in recent years, as has interest in the role of biomass burning as a driver of and response to past environmental change. Several approaches, including decomposition of charcoal time series, multi-proxy studies, multi-site comparisons within regions, and composite records at regional-to-global scales have been used to characterize fire regimes and test hypotheses regarding the relative influences of climate, vegetation, and humans on fire regimes. These insights from the past help provide important context for efforts to manage fire, conserve biodiversity, and restore ecosystems (Willis et al., 2005). Full realization of the sedimentary charcoal potential, however, requires continued research into charcoal taphonomy, and the relationship between fire activity and charcoal accumulation rates in different depositional settings (i.e., large vs. small lakes; mountainous vs. flat regions; forested ecosystems vs. grasslands). In addition, laboratory and analytical techniques employed in charcoal analysis and interpretation should continually be reviewed to validate methodological assumptions, assure replicability, and quantify uncertainties. There is also a need to interpret charcoal data in ways that are more closely comparable to simulations of fire. For example, relative abundances of charcoal in paleofire records are less useful than estimates of area burned, fire intensity or carbon emissions for data-model comparisons.

Recent advances in ecological modeling present opportunities to reduce some of these uncertainties. For example, simulations of charcoal accumulation rates have helped fill gaps in our understanding of charcoal taphonomy, which is an important step in establishing a two-way flow of information between disciplines. Modeling approaches can also be used to interpolate—spatially and temporally—between charcoal records. Furthermore, fire simulation results may be considered as independent evidence to support ecological dynamics inferred from charcoal records.

To date, paleoecological records have been used in model validation, but it is possible to integrate paleoecological data in all phases of model development and testing. Such integration opens the door to a new generation of hypotheses in fire research and a more realistic understanding of fire and its drivers at different spatial and temporal scales. Realistic representations of climate-vegetation-fire linkages can be interrogated with resource planning goals, inasmuch as they provide a sound basis for identifying trends, mechanisms and thresholds under different socio-environmental conditions (Dearing et al., 2012).

### Conflict of interest statement

The authors declare that the research was conducted in the absence of any commercial or financial relationships that could be construed as a potential conflict of interest.
